# Hostile Attribution Bias Mediates the Relationship Between Structural Variations in the Left Middle Frontal Gyrus and Trait Angry Rumination

**DOI:** 10.3389/fpsyg.2018.00526

**Published:** 2018-04-11

**Authors:** Yueyue Wang, Wenfeng Zhu, Mingyue Xiao, Qin Zhang, Yufang Zhao, Hao Zhang, Xu Chen, Yong Zheng, Ling-Xiang Xia

**Affiliations:** ^1^Faculty of Psychology, Southwest University, Chongqing, China; ^2^Key Laboratory of Cognition and Personality, Ministry of Education, Southwest University, Chongqing, China

**Keywords:** angry rumination, hostile attribution bias, left middle frontal gyrus, voxel-based morphometry, mediation

## Abstract

Angry rumination is a common mental phenomenon which may lead to negative social behaviors such as aggression. Although numerous neuroimaging studies have focused on brain area activation during angry rumination, to our knowledge no study has examined the neuroanatomical and cognitive mechanisms of this process. In this study, we conducted a voxel-based morphometry analysis, using a region of interest analysis to identify the structural and cognitive mechanisms underlying individual differences in trait angry rumination (as measured by the Angry Rumination Scale) in a sample of 82 undergraduate students. We found that angry rumination was positively correlated with gray matter density in the left middle frontal gyrus (left-MFG), which is implicated in inhibition control, working memory, and emotional regulation. The mediation analysis further revealed that hostile attribution bias (as measured by the Social Information Processing–Attribution Bias Questionnaire) acted as a cognitive mechanism underlying the positive association between the left-MFG gray matter density and trait angry rumination. These findings suggest that hostile attribution bias may contribute to trait angry rumination, while the left-MFG may play an important role in the development of hostile attribution bias and trait angry rumination. The study reveals the brain mechanisms of trait angry rumination and plays a role in revealing the cognitive mechanisms of the development of trait angry rumination.

## Introduction

Rumination is a common mental phenomenon, particularly for those with mental illness. Depressed individuals may have chronic, repetitive thoughts about why he or she feels bad, while anxious people often worry that something bad will happen (Borkovec et al., 1998; Nolenhoeksema et al., 2008). Martin and Tesser (1996) defined rumination as “a class of conscious thoughts that revolve around a common instrumental theme and that recur in the absence of immediate environmental demands requiring the thoughts” (p. 7). When the theme of rumination is an anger-inducing event, angry rumination occurs (Sukhodolsky et al., 2001; Denson et al., 2006). Specifically, angry rumination refers to repetitive thoughts about a personally meaningful event that induced anger, accompanied by angry feelings or thoughts about revenge (Sukhodolsky et al., 2001; Denson et al., 2006; Denson, 2013).

Angry rumination is a complex psychological phenomenon involving such processes as social cognition, negative affect, and emotion regulation. It may occur as state angry rumination or as trait angry rumination. In the past decade, research on angry rumination has been conducted in several fields, including personality psychology, social psychology, and neuroscience. Previous behavioral studies have shown that angry rumination is associated with negative outcomes such as aggressive behavior (Denson et al., 2011; Pedersen et al., 2011; Denson, 2013), negative emotion, and depressive symptoms and episodes (Segerstrom et al., 2000; Bushman et al., 2005).

In neuroscience, a number of studies have examined the relationship between angry rumination and brain activity, and suggest that the prefrontal cortex (PFC), amygdala, and thalamus may be the primary brain area related to angry rumination. For example, brain areas in the PFC and limbic system become active and functionally connected during angry rumination (Denson, 2013). Denson et al. (2009) found that during angry rumination, people recruited brain regions involved in the intensity of negative affect, as well as those involved in emotional regulation, such as the lateral and medial PFC. One recent study demonstrated that angry rumination may recruit brain regions involved with negative emotions and arousal, such as the amygdala and thalamus (Fabiansson et al., 2012). Additionally, functional magnetic resonance imaging (fMRI) studies have suggested that angry rumination is associated with activity in a number of frontal regions (e.g., medial frontal gyrus, dorsal anterior cingulate, lateral middle frontal gyrus) (Ray et al., 2005; Kelley et al., 2013).

Trait angry rumination is a kind of personality trait, which is relatively stable across situations and times, reflect a behavior model; while state angry rumination is a kind of behavior response that is not stable and is determined by specific situations. However, such studies (Denson et al., 2009; Fabiansson et al., 2012) have focused on state angry rumination, which researchers induced by a provocation-focused rumination task or some other experimental protocol. To our knowledge, no study has examined the neural basis of trait angry rumination, which is measured through questionnaires. The present study focuses on the neural structural basis of trait angry rumination.

The neuroimaging studies discussed here have consistently indicated that the PFC, which is involved in cognitive–emotional functions such as emotional regulation (Ochsner and Gross, 2005; Kalisch, 2009) and executive functioning (Alvarez and Emory, 2006; Blumenfeld and Ranganath, 2006), and the amygdala and thalamus, which are involved in emotional experiences (Lanteaume et al., 2007), may play important roles in angry ruminative activity. Therefore, it is reasonable to presume that they are also active in the development of trait angry rumination. Additionally, we hoped to identify which specific regions of the PFC are associated with trait angry rumination by examining the relationship between angry rumination and neural structure. In short, we assume that trait angry rumination may be associated with the regional gray matter (GM) density of certain prefrontal cortical regions (e.g., the middle frontal gyrus and medial frontal gyrus) related to inhibition control, social perception, working memory, and emotional regulation, as well as the amygdala and thalamus, which implicated in an evaluation of affective stimuli (Ray et al., 2005; Blumenfeld and Ranganath, 2006; Vã Llm et al., 2006; Denson et al., 2009; Denson, 2013).

Prior studies (Kong et al., 2015; Li et al., 2015; Dolcos et al., 2016) have suggested that certain psychological variables may mediate the relationship between brain structure and personality traits. Here, we explore the mechanism mediating the density of the PFC and trait angry rumination. For several reasons, we have identified hostile attribution bias as a candidate for this mechanism.

First, hostile attribution bias may influence trait angry rumination. Hostile attribution bias is a kind of interpretation bias in which individuals are more likely to interpret ambiguous situations as hostile than benign (Epps and Kendall, 1995; Wilkowski and Robinson, 2010). Negative interpretations of ambiguity are a potential cause of rumination (Hertel et al., 2014). Furthermore, previous studies have indicated that interpretation bias affects angry rumination (Lam et al., 2003). Specifically, studies of hostile attribution bias have indicated that hostile information bias automatically captures attention (Pratto and John, 1991; Robinson, 1998); this capture of attention toward hostile information in turn leads, quite naturally, to rumination upon such information (Wilkowski and Robinson, 2008). Prolonged allocation of attention to anger-inducing events is one of the key features of angry rumination; thus, individuals who tend to make hostile attributions may be more susceptible to ruminate in response to anger-inducing events. In addition, hostile attribution bias is a response bias in ambiguous situations: the ambiguity of the situation induces individual to think about it repetitively. Finally, some studies suggest that hostile interpretations are involved in the elicitation of anger (Wilkowski and Robinson, 2010). In sum, hostile attribution bias may predict trait angry rumination.

Second, the PFC may also be associated with hostile attribution bias. The neural processes involved in the ability to understand others’ intentions have been studied from multiple neuroscience perspectives, such as attribution of intention to others and theory of mind (Frith and Frith, 2006; Gagnon et al., 2016). Regions of the PFC are known to contribute to the development of attribution of intention to others (Vã Llm et al., 2006). For example, the dorsal medial PFC becomes active when people make attributions, or inferences about the mental states of others (Harris et al., 2005; Krämer et al., 2007). Some fMRI studies have shown that attributions about others’ thoughts and intentions is associated with activity in the mPFC (Brunet et al., 2000; Spiers and Maguire, 2006; Vollm et al., 2006). Finally, attribution about others has been consistently linked to neural networks within the mPFC (Frith and Frith, 2006). In summary, previous studies have shown that the neural processes involved in understanding others’ intentions might be correlated with activity in the PFC (e.g., the mPFC and the dorsal mPFC) (Harris et al., 2005; Frith and Frith, 2006; Krämer et al., 2007). Since hostile attribution bias also involves assessment of others’ intentions, we assume that it may have a similar neural basis to mindreading about others. Hostile attribution bias may be related to regional GM density of the PFC (Macrae et al., 2004; Ochsner et al., 2004).

The aim of the study was twofold: firstly, to identify the structural and cognitive mechanisms underlying individual differences in trait angry rumination, and, secondly, to examine the mediating role of hostile attribution bias in the relationship between PFC density and trait angry rumination. We performed both voxel-based morphometry (VBM) and a regions of interest (ROI) analysis. According to previous findings by neuroscientists on angry rumination (Ray et al., 2005; Denson et al., 2009; Denson, 2013), ROI selection was based on previous related studies. We hypothesized that individual difference in trait angry rumination would be associated with density in the PFC, amygdala, and thalamus. Finally, given the association between trait angry rumination and hostile attribution bias, as well as the crucial role of the PFC in hostile attribution bias, we hypothesized that hostile attribution bias would be able to mediate the relationship between brain structure and trait angry rumination.

## Materials and Methods

### Participants

The study sample comprised 82 healthy volunteers (32 men; mean age = 21.03, *SD* = 1.9; age range: 18–27 years) participated in the study. All participants were undergraduate students from Southwest University in China. Participants were instructed to complete paper–pencil questionnaires and have fMRI scans. Every participant was right-handed, and each completed a questionnaire. All participants signed an informed consent document prior to the experiment and were compensated with money in the final of the study.

### Measure of Angry Rumination

The Angry Rumination Scale (ARS) (Sukhodolsky et al., 2001) is a 19-item questionnaire measuring the tendency to ruminate on anger-inducing events. It contains four principal scales: understanding of causes, angry afterthoughts, thoughts of revenge, and angry memories. Items were rated on a four-point Likert scale (1 = almost never; 4 = almost always). We used the Chinese version of the scale, which has been shown in previous studies to have good reliability, good validity, and good criterion-related validity (Maxwell et al., 2007; Luo and Liu, 2017). Furthermore, we conducted a confirmatory factor analysis (CFA) of a large sample (*N* = 898). These results revealed that the model fit indices had acceptably fit the data (RMSEA = 0.08, CFI = 0.993, TLI = 0.978, SRMR = 0.014). The results indicated that the Chinese version of the ARS had good construction validity. The Cronbach’s alpha in this sample was 0.95.

### Measure of Hostile Attribution Bias

Hostile attribution bias was assessed using the Hostile Attribution Bias subscale from the Social Information Processing–Attribution Bias Questionnaire (SIP–ABQ) (Coccaro et al., 2009). This questionnaire contains eight scenarios, each describing a situation with a negative outcome and includes a character whose intentions are ambiguous. Participants were asked to rate the likelihood of two hostile explanations (e.g., “My friend wanted to expose my secret”; “My friend wanted me to feel stupid for asking to keep my secret”) per scenario on a range from 0 (not at all likely) to 3 (very likely). The sum scores of the 16 items represent the degree of hostile attribution bias. In the present study, we used the Chinese version of Hostile Attribution Bias subscale, which had been tested via another large sample (*N* = 913) by one of the authors. The CFA of that sample revealed that the one factor model fit with the data well, RMSEA = 0.03, CFI = 0.999, TLI = 0.997, SRMR = 0.006. The results indicated that the Chinese version of Hostile Attribution Bias subscale had good construct validity. Furthermore, the test–retest reliability was 0.61 (6-month interval) in a large sample (*N* = 942). The Cronbach’s alpha in this sample was 0.92.

### MRI Data Acquisition

Scanning was performed on a 3.0-T Siemens Trio MRI scanner (Siemens Medical, Erlangen, Germany). MRI structural images were acquired using a magnetization-prepared rapid gradient echo (MP-RAGE) sequence (repetition time [TR] = 1900 ms; echo time [TE] = 2.52 ms; flip angle = 9 degrees; inversion time [TI] = 900 ms; thickness = 1.0 mm; resolution matrix = 256 × 256; slices = 176; voxel size = 1 mm × 1 mm × 1 mm).

### Data Preprocessing

Structural magnetic Resonance images were performed with SPM8 (Welcome Department of Cognitive Neurology, London, United Kingdom). First, image quality was screened in SPM8 for artifacts or gross anatomical abnormalities. Second, in order to obtain better registration, we corrected the anterior-posterior commissure (AC-PC) line. Third, we used the new segmentation in SPM8 to segment the images into GM, white matter (WM), and cerebrospinal fluid (Ashburner and Friston, 2005). Fourth, we administered the Diffeomorphic Anatomical Registration Through Exponential Lie Algebra (DARTEL) method of registration, normalization, and modulation (Ashburner, 2007). DARTEL registration involves repeatedly computing the specific template based on average tissue probability maps from all participants, then wrapping each participant’s maps into the specific template to improve alignment. To achieve more accurate registration, this process was repeated until an ideal template was generated. In order to preserve the volume of tissue in each structure after warping, the image intensity of every voxel was modulated by the Jacobian determinants. Gray matter images were rigidly aligned and resampled to 1 mm × 1 mm × 1 mm. The registered images were normalized to Montreal Neurological Institute (MNI) space. Finally, the modulated images were smoothed to a 10-mm Full Width at Half Maximum Gaussian kernel in order to improve signal-to-noise ratio.

### Statistical Analysis

#### Correlation Analyses

All behavioral variables and brain regions were analyzed using SPSS 22.0 software. Descriptive statistics and Pearson correlation were assessed to examine the relationship between GM density and personality traits score.

#### Region of Interest Definition (ROI)

Based on previous studies (Ray et al., 2005; Denson et al., 2009; Kelley et al., 2013) and our hypothesis, the ROIs were selected because of previous evidence, which included the AAL Library templates (Tzouriomazoyer et al., 2002), that particular brain areas—including the PFC, the amygdala, and the thalamus—may be related to angry rumination. We then used the Wake Forest University (WFU) Pick Atlas (Maldjian et al., 2003) to delineate these regions and create image masks that were used, according to Small Volume Correction (SVC) procedures, to restrict voxel-wise analyses to the ROI.

#### MRI Data Analysis

Statistical analyses of MRI data were conducted using SPM8 software. During this analysis, we used a multiple linear regression to investigate the anatomical correlates of individual differences in angry rumination. In the process of multiple linear regression, angry rumination score was used as the variable of interest, whereas the age, gender, and total GM density were entered as the covariates of no interest. In the analyses, the statistical significance of ROI analysis was set at *P* < 0.05 at cluster level and corrected using non-stationary cluster correction (Hayasaka et al., 2004) with a voxel-wise level of *P* < 0.001.

In order to define the significantly correlated region, we first saved the results of the significant cluster. We then extracted the ROI signals from each participant’s data using REX toolbox (Massachusetts Institute of Technology, Cambridge, MA, United States).

#### Mediation Analysis

In order to determine whether hostile attribution bias could mediate the relationship between regional GM density and angry rumination, we conducted a mediation analysis. Mediation analyses were conducted using the PROCESS macro in SPSS (Hayes, 2013). In this study, angry rumination was the dependent variable, hostile attribution bias was the mediating variable, and the GM density of regions of the brain associated with angry rumination was the independent variable. The PROCESS macro used bootstrapped sampling to compute the indirect mediation effect. In this study, we drew 5,000 bootstrapped samples and bias-corrected 95% confidence intervals. The indirect effect of the independent variable on angry rumination through hostile attribution bias was significant when confidence intervals did not include zero.

## Results

### Structural Imaging Results

**FIGURE 1:**
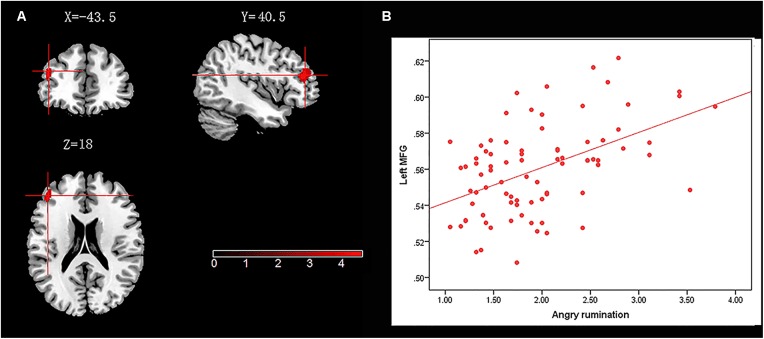
Brain regions that positively correlated with angry rumination. **(A)** The left middle frontal gyrus (left-MFG) with gray matter (GM) density was positively correlated with angry rumination. **(B)** Scatter plots depicting correlations between angry rumination and left-MFG.

We administered a multiple regression model to investigate the association between neural structure (measured in terms of GM density) and trait angry rumination. Age, gender, and total GM density were entered as covariates of no interest and regressed out. In the process, we created image mask (including entire PFC, amygdala, and thalamus) that were used to restrict voxel-wise analyses to the ROI. ROI analyses revealed that angry rumination was positively associated with GM density in a cluster that mainly included an area in the left middle frontal gyrus (left-MFG) (*x* = -43.5, *y* = 40.5, *z* = 18, cluster size = 287, voxels, *t* = 4.62, *p* < 0.05; see **Table 2** and **Figure 1** for the peak coordination regions).

### Correlation Analyses Results

Descriptive statistics and Pearson correlation coefficients for behavioral variables and brain regions were presented in **Table 1**; they show that hostile attribution bias was positively correlated with trait angry rumination (*r* = 0.41, *p* < 0.01), indicating that participants with high hostile attribution bias may also have high trait angry rumination. On the other hand, we observed a significant positive correlation between hostile attribution bias and left-MFG (*r* = 0.27, *p* < 0.05).

**Table 1:** Descriptive statistics and correlations between behavioral variables and brain regions (*N* = 82; men = 32).

Measure	*M*	*SD*	Range	1	2	3
(1) Angry rumination	1.96	0.62	[1.05,3.79]	1		
(2) Hostile attribution bias	1.07	0.51	[0.06,2.13]	0.41^∗∗^	1	
(3) Left-MFG	0.56	0.02	[0.51,0.62]	0.49^∗∗^	0.27^∗^	1

*^∗^*p* < 0.05; ^∗∗^*p* < 0.01.*

**Table 2:** Summary of the gray matter density associations with angry rumination.

Brain region	H	MNI coordinate *x y z*	Peak *t*-value	Cluster size (mm^3^)
Positive correlation left-MFG	L	-43.5 40.5 18	4.62	287

*left-MFG, left middle frontal gyrus; H, hemisphere.*

### Mediation Results

**FIGURE 2:**
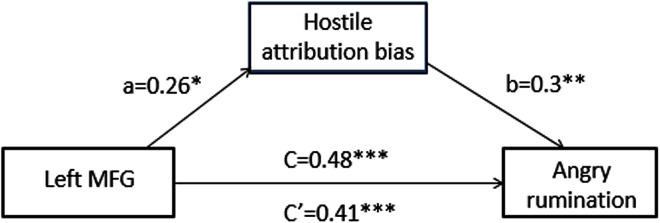
Hostile attribution bias mediates the association between brain structure and angry rumination: path diagram of the mediation analysis demonstrating that the left-MFG affected individuals’ angry rumination through hostile attribution bias. Total effect (c): *b* = 0.48, *SE* = 0.09, *p* < 0.001. Direct effect (c′): *b* = 0.41, *SE* = 0.09, *p* < 0.001. Indirect effect (ab): *b* = 0.08, Boot *SE* = 0.04, 95%CI = [0.02, 0.18]. ^∗^*p* < 0.05; ^∗∗^*p* < 0.01; ^∗∗∗^*p* < 0.001.

In order to assess our hypothesis, we first examined the relationships between hostile attribution bias and GM density in a cluster that correlated with trait angry rumination. As expected, we found that hostile attribution bias was positively correlated with a left-MFG cluster (*r* = 0.27, *p* < 0.05). These results indicate that trait angry rumination, hostile attribution bias, and left-MFG density are closely linked. In order to examine whether hostile attribution bias mediated the relationship between trait angry rumination and left-MFG density, mediation analysis was performed using the PROCESS macro in SPSS 22.0 (using PROCESS model 4, basic mediation). As illustrated in **Figure 2**, hostile attribution bias significantly mediated the relationship between left-MFG density (95% confidence interval = [0.02, 0.18]) and trait angry rumination.

## Discussion

The neural basis of angry rumination has received considerable attention (Ray et al., 2005; Fabiansson et al., 2012), but no research has focused on the topic of trait angry rumination. In this study, we used VBM to explore the association between brain structure and trait angry rumination in healthy individuals. We discovered that greater density in the left-MFG is associated with higher trait angry rumination. Our results are in some ways consistent with the neural basis for state angry rumination. Several studies have shown that state angry rumination was associated with activation of the PFC, which are implicated in the evaluation of affective stimuli (Ray et al., 2005; Denson et al., 2009; Fabiansson et al., 2012; Denson, 2013; Kelley et al., 2013). Our study lends further support to these results with the finding that the left-MFG was also associated with trait angry rumination, suggesting that the left-MFG plays an important role in both state and trait angry rumination.

To our knowledge, this is the first study to reveal the neural basis of trait angry rumination. Activation of the left-MFG has been consistently found to be associated with working memory (Zhang et al., 2003), processing social information and social perception (Vã Llm et al., 2006), memory retrieval (Tulving et al., 1994), processing emotional stimuli (Bermpohl et al., 2006) and emotional regulation (Ochsner and Gross, 2005). These cognitive functions of the left-MFG are consistent with the cognitive factors of rumination or trait rumination. Furthermore, this region is also found to be proposed as the cortical focus for both the storage and the processing components of working memory in the human brain (Leung et al., 2002), which are crucial for the formation of angry rumination. In addition, trait rumination is related to the ability to ignore distracting information while maintaining relevant information (Joormann et al., 2010; Zetsche and Joormann, 2011). The positive correlation between trait angry rumination and left-MFG indicated that the higher density in left-MFG might help individuals to regulate their angry emotions, maintain anger-inducing events in working memory, thus leading to higher levels of angry rumination.

As expected, GM density in the left-MFG is also associated with trait hostile attribution bias. The left-MFG is associated with processing social information and social perception (Vã Llm et al., 2006). Presumably, hostile attribution bias might induce some particular patterns of cognitive processing associated with both the automatic capture of and rumination upon hostile information (Lam et al., 2003; Hertel et al., 2014). Furthermore, hostile attribution bias is a kind of interpretation bias when it occurs in ambiguous situations (Epps and Kendall, 1995). Individuals who tend toward hostile attribution biases should have some hostile related knowledge structures and schemas stored in their memories, which they retrieve when explaining the social information arising from these ambiguous situations. The left-MFG was found to be associated with memory retrieval (Tulving et al., 1994), which may be crucial for the hostile attribution process in ambiguous situations. Based on this evidence, hostile attribution bias may recruit the left-MFG, which is related to the functions of social perception and memory retrieval.

Our behavioral data show that the scores for hostile attribution bias were strongly and significantly correlated with trait angry rumination. This result is similar to those of previous studies that indicated that interpretation bias—especially hostile attribution bias—is strongly related to angry rumination (Wilkowski and Robinson, 2008; Hertel et al., 2014). Previous findings also show that individuals who exhibit hostile attribution bias prioritize hostility-inducing information, and that hostility-inducing information can elicit anger. Consequently, chronically prolonged rumination on hostile information and anger may promote the development of trait angry rumination. In addition, hostile attribution bias is a response bias that occurs in ambiguous situations. The ambiguity of these situations induces individuals to repeat angry mental cues. This repetitive activity that may also promote the development of trait angry rumination. It therefore seems evident that hostile attribution bias might play an important role in shaping trait angry rumination.

The results presented in **Figure 2** demonstrate that hostile attribution bias mediated the association between left-MFG density and trait angry rumination. This finding sheds light on the underlying cognitive mechanisms of trait angry rumination with respect to the effects of GM density in the left-MFG. Results of this study suggest that the neuroanatomical characteristics may influence cognitive habits and preferences, such as hostile attribution bias, which in turn affect the development of cognition traits such as trait angry rumination.

Regarding this study’s limitations, the sample included only young, highly educated adults; it is unclear whether our results would be replicated in other samples. Future studies could focus on replicating these results in other samples, such as adults from the wider community. Second, our research utilized a cross-sectional design, which is unable to demonstrate causal relationships; therefore, prospective longitudinal studies are needed to explore potential causally related associations between the left-MFG density, hostile attribution bias, and trait angry rumination.

## Ethics Statement

This study was carried out in accordance with the recommendations of ‘the guidelines of the International Committee of Medical Journal Editors, the Ethics Committee of Southwest University of China’ with written informed consent from all subjects in accordance with the Declaration of Helsinki. The protocol was approved by the ‘the Ethics Committee of Southwest University of China.’

## Author Contributions

YW, L-XX, and WZ drafted the manuscript. YW, YuZ, HZ, XC, MX, QZ, and YoZ did acquisition and analysis. All authors approved the final version of the manuscript for submission and contributed to the development of the studies.

## Conflict of Interest Statement

The authors declare that the research was conducted in the absence of any commercial or financial relationships that could be construed as a potential conflict of interest.

## References

[B1] Alvarez J. A. Emory E. (2006). Executive function and the frontal lobes: a meta-analytic review. *Neuropsychol. Rev.* 16 17–42. 10.1007/s11065-006-9002-x 16794878

[B2] Ashburner J. (2007). A fast diffeomorphic image registration algorithm. *Neuroimage* 38 95–113. 10.1016/j.neuroimage.2007.07.007 17761438

[B3] Ashburner J. Friston K. J. (2005). Unified segmentation. *Neuroimage* 26 839–851. 10.1016/j.neuroimage.2005.02.018 15955494

[B4] Bermpohl F. Pascualleone A. Amedi A. Merabet L. B. Fregni F. Gaab N. (2006). Attentional modulation of emotional stimulus processing: an fMRI study using emotional expectancy. *Hum. Brain Mapp.* 27 662–677. 10.1002/hbm.20209 16317710 PMC6871342

[B5] Blumenfeld R. S. Ranganath C. (2006). Dorsolateral prefrontal cortex promotes long-term memory formation through its role in working memory organization. *J. Neurosci.* 26 916–925. 10.1523/JNEUROSCI.2353-05.2006 16421311 PMC6675388

[B6] Borkovec T. D. Ray W. J. Stober J. (1998). Worry: a cognitive phenomenon intimately linked to affective, physiological, and interpersonal behavioral processes. *Cogn. Ther. Res.* 22 561–576. 10.1023/A:1018790003416

[B7] Brunet E. Sarfati Y. Hardy-Baylé M. C. Decety J. (2000). A PET investigation of the attribution of intentions with a nonverbal task. *Neuroimage* 11 157–166. 10.1006/nimg.1999.0525 10679187

[B8] Bushman B. J. Bonacci A. M. Pedersen W. C. Vasquez E. A. Miller N. (2005). Chewing on it can chew you up: effects of rumination on triggered displaced aggression. *J. Pers. Soc. Psychol.* 88 969–983. 10.1037/0022-3514.88.6.969 15982116

[B9] Coccaro E. F. Noblett K. L. Mccloskey M. S. (2009). Attributional and emotional responses to socially ambiguous cues: validation of a new assessment of social/emotional information processing in healthy adults and impulsive aggressive patients. *J. Psychiatr. Res.* 43 915–925. 10.1016/j.jpsychires.2009.01.012 19345371

[B10] Denson T. F. (2013). The multiple systems model of angry rumination. *Pers. Soc. Psychol. Rev.* 17 103–123. 10.1177/1088868312467086 23175519

[B11] Denson T. F. Pedersen W. C. Friese M. Hahm A. Roberts L. (2011). Understanding impulsive aggression: angry rumination and reduced self-control capacity are mechanisms underlying the provocation-aggression relationship. *Pers. Soc. Psychol. Bull.* 37 850–862. 10.1177/0146167211401420 21421767

[B12] Denson T. F. Pedersen W. C. Miller N. (2006). The displaced aggression questionnaire. *J. Pers. Soc. Psychol.* 90 1032–1051. 10.1037/0022-3514.90.6.1032 16784350

[B13] Denson T. F. Pedersen W. C. Ronquillo J. Nandy A. S. (2009). The angry brain: neural correlates of anger, angry rumination, and aggressive personality. *J. Cogn. Neurosci.* 21 734–744. 10.1162/jocn.2009.21051 18578600

[B14] Dolcos S. Hu Y. Iordan A. Moore M. Dolcos F. (2016). Optimism and the brain: trait optimism mediates the protective role of the orbitofrontal cortex gray matter volume against anxiety. *Soc. Cogn. Affect. Neurosci.* 11 263–271. 10.1093/scan/nsv106 26371336 PMC4733335

[B15] Epps J. Kendall P. C. (1995). Hostile attribution bias in adults. *Cogn. Ther. Res.* 19 159–178. 10.1007/BF02229692

[B16] Fabiansson E. C. Denson T. F. Moulds M. L. Grisham J. R. Schira M. M. (2012). Don’t look back in anger: neural correlates of reappraisal, analytical rumination, and angry rumination during recall of an anger-inducing autobiographical memory. *Neuroimage* 59 2974–2981. 10.1016/j.neuroimage.2011.09.078 22015853

[B17] Frith C. D. Frith U. (2006). How we predict what other people are going to do. *Brain Res.* 1079 36–46. 10.1016/j.brainres.2005.12.126 16513098

[B18] Gagnon J. Aubin M. Emond F. C. Derguy S. Bessette M. Jolicoeur P. (2016). Neural mechanisms underlying attribution of hostile intention in nonaggressive individuals: an ERP study. *Int. J. Psychophysiol.* 110 153–162. 10.1016/j.ijpsycho.2016.08.007 27543324

[B19] Harris L. T. Todorov A. Fiske S. T. (2005). Attributions on the brain: neuro-imaging dispositional inferences, beyond theory of mind. *Neuroimage* 28 763–769. 10.1016/j.neuroimage.2005.05.021 16046148

[B20] Hayasaka S. Phan K. L. Liberzon I. Worsley K. J. Nichols T. E. (2004). Nonstationary cluster-size inference with random field and permutation methods. *Neuroimage* 22 676–687. 10.1016/j.neuroimage.2004.01.041 15193596

[B21] Hayes A. F. (2013). Introduction to mediation, moderation, and conditional process analysis: a regression-based approach. *J. Educ. Meas.* 51 335–337. 10.1080/13557858.2017.1315056 28385036

[B22] Hertel P. Mor N. Ferrari C. Hunt O. Agrawal N. (2014). Looking on the dark side: rumination and cognitive-bias modification. *Clin. Psychol. Sci.* 2 714–726. 10.1177/2167702614529111

[B23] Joormann J. Nee D. E. Berman M. G. Jonides J. Gotlib I. H. (2010). Interference resolution in major depression. *Cogn. Affect. Behav. Neurosci.* 10 21–33. 10.4088/PCC.16m01949 20233953 PMC2845922

[B24] Kalisch R. (2009). The functional neuroanatomy of reappraisal: time matters. *Neurosci. Biobehav. Rev.* 33 1215–1226. 10.1016/j.neubiorev.2009.06.003 19539645

[B25] Kelley N. J. Hortensius R. Harmonjones E. (2013). When anger leads to rumination: induction of relative right frontal cortical activity with transcranial direct current stimulation increases anger-related rumination. *Psychol. Sci.* 24 475–481. 10.1177/0956797612457384 23449843

[B26] Kong F. Hu S. Xue S. Song Y. Liu J. (2015). Extraversion mediates the relationship between structural variations in the dorsolateral prefrontal cortex and social well-being. *Neuroimage* 105 269–275. 10.1016/j.neuroimage.2014.10.062 25449749

[B27] Krämer U. M. Jansma H. Tempelmann C. Münte T. F. (2007). Tit-for-tat: the neural basis of reactive aggression. *Neuroimage* 38 203–211. 10.1016/j.neuroimage.2007.07.029 17765572

[B28] Lam D. Smith N. Checkley S. Rijsdijk F. Sham P. (2003). Effect of neuroticism, response style and information processing on depression severity in a clinically depressed sample. *Psychol. Med.* 33 469–479. 10.1017/S0033291702007304 12701667

[B29] Lanteaume L. Khalfa S. Régis J. Marquis P. Chauvel P. Bartolomei F. (2007). Emotion induction after direct intracerebral stimulations of human amygdala. *Cereb. Cortex* 17 1307–1313. 10.1093/cercor/bhl041 16880223

[B30] Leung H. C. Gore J. C. Goldman-Rakic P. S. (2002). Sustained mnemonic response in the human middle frontal gyrus during on-line storage of spatial memoranda. *J. Cogn. Neurosci.* 14 659–671. 10.1162/08989290260045882 12126506

[B31] Li W. Li X. Huang L. Kong X. Yang W. Wei D. (2015). Brain structure links trait creativity to openness to experience. *Soc. Cogn. Affect. Neurosci.* 10 191–198. 10.1093/scan/nsu041 24603022 PMC4321617

[B32] Luo Y. L. Liu Y. B. (2017). An examination of the reliability and validity of the Chinese version of the anger rush thinking scale. *Chin. J. Clin. Psychol.* 25 667–670. 10.16128/j.cnki.1005-3611.2017.04.017

[B33] Macrae C. N. Moran J. M. Heatherton T. F. Banfield J. F. Kelley W. M. (2004). Medial prefrontal activity predicts memory for self. *Cereb. Cortex* 14 647–654. 10.1093/cercor/bhh025 15084488

[B34] Maldjian J. A. Laurienti P. J. Kraft R. A. Burdette J. H. (2003). An automated method for neuroanatomic and cytoarchitectonic atlas-based interrogation of fMRI data sets. *Neuroimage* 19 1233–1239. 10.1016/S1053-8119(03)00169-1 12880848

[B35] Martin L. L. Tesser A. (1996). “Some ruminative thoughts,” in *Advances in social cognition, Vol. 9 Ruminative Thoughts* ed. Wyer R. S. Jr. (Hillsdale, NJ: Lawrence Erlbaum Associates, Inc.) 1–47.

[B36] Maxwell J. P. Moores E. Chow C. C. F. (2007). Anger rumination and self-reported aggression amongst British and Hong Kong Chinese athletes: a cross cultural comparison. *Int. J. Sport Exerc. Psychol.* 5 9–27. 10.1080/1612197X.2008.9671810

[B37] Nolenhoeksema S. Wisco B. E. Lyubomirsky S. (2008). Rethinking rumination. *Perspect. Psychol. Sci. A J. Assoc. Psychol. Sci.* 3 400–424. 10.1111/j.1745-6924.2008.00088.x 26158958

[B38] Ochsner K. N. Gross J. J. (2005). The cognitive control of emotion. *Trends Cogn. Sci.* 9 242–249. 10.1016/j.tics.2005.03.010 15866151

[B39] Ochsner K. N. Knierim K. Ludlow D. H. Hanelin J. Ramachandran T. Glover G. (2004). Reflecting upon feelings: an fMRI study of neural systems supporting the attribution of emotion to self and other. *J. Cogn. Neurosci.* 16 1746–1772. 10.1162/0898929042947829 15701226

[B40] Pedersen W. C. Denson T. F. Goss R. J. Vasquez E. A. Kelley N. J. Miller N. (2011). The impact of rumination on aggressive thoughts, feelings, arousal, and behaviour. *Br. J. Soc. Psychol.* 50 281–301. 10.1348/014466610X515696 21545459

[B41] Pratto F. John O. P. (1991). Automatic vigilance: the attention-grabbing power of negative social information. *J. Pers. Soc. Psychol.* 61 380–391. 10.1037/0022-3514.61.3.380 1941510

[B42] Ray R. D. Ochsner K. N. Cooper J. C. Robertson E. R. Gabrieli J. D. E. Gross J. J. (2005). Individual differences in trait rumination and the neural systems supporting cognitive reappraisal. *Cogn. Affect. Behav. Neurosci.* 5 156–168. 10.3758/CABN.5.2.156 16180622

[B43] Robinson M. D. (1998). Running from William James’ Bear: a review of preattentive mechanisms and their contributions to emotional experience. *Cogn. Emot.* 12 667–696. 10.1080/026999398379493 16144959

[B44] Segerstrom S. C. Tsao J. C. I. Alden L. E. Craske M. G. (2000). Worry and rumination: repetitive thought as a concomitant and predictor of negative mood. *Cogn. Ther. Res.* 24 671–688. 10.1023/A:1005587311498

[B45] Spiers H. J. Maguire E. A. (2006). Spontaneous mentalizing during an interactive real world task: an fMRI study. *Neuropsychologia* 44 1674–1682. 10.1016/j.neuropsychologia.2006.03.028 16687157

[B46] Sukhodolsky D. G. Golub A. Cromwell E. N. (2001). Development and validation of the anger rumination scale. *Pers. Individ. Diff.* 31 689–700. 10.1016/S0191-8869(00)00171-9

[B47] Tulving E. Kapur S. Markowitsch H. J. Craik F. I. Habib R. Houle S. (1994). Neuroanatomical correlates of retrieval in episodic memory: auditory sentence recognition. *Proc. Natl. Acad. Sci. U.S.A.* 91 2012–2015. 10.1073/pnas.91.6.2012 8134341 PMC43299

[B48] Tzouriomazoyer N. Landeau B. Papathanassiou D. Crivello F. Etard O. Delcroix N. (2002). Automated anatomical labeling of activations in SPM using a macroscopic anatomical parcellation of the MNI MRI single-subject brain. *Neuroimage* 15 273–289. 10.1006/nimg.2001.0978 11771995

[B49] Vã Llm B. Taylor A. Richardson P. Corcoran R. Stirling J. Mckie S. (2006). Neural correlates of theory of mind and empathy: a functional magnetic resonance imaging study in a nonverbal task. *Neuroimage* 29 90–98. 10.1016/j.neuroimage.2005.07.022 16122944

[B50] Vollm B. A. Taylor A. P. Corcoran R. Stirling J. Mckie S. Deakin J. F. (2006). Neuronal correlates of theory of mind and empathy: a functional magnetic resonance imaging study in a nonverbal task. *Neuroimage* 29 90–98. 10.1016/j.neuroimage.2005.07.022 16122944

[B51] Wilkowski B. M. Robinson M. D. (2008). The cognitive basis of trait anger and reactive aggression: an integrative analysis. *Pers. Soc. Psychol. Rev.* 12 3–21. 10.1177/1088868307309874 18453470

[B52] Wilkowski B. M. Robinson M. D. (2010). The anatomy of anger: an integrative cognitive model of trait anger and reactive aggression. *J. Pers.* 78 9–38. 10.1111/j.1467-6494.2009.00607.x 20433611

[B53] Zetsche U. Joormann J. (2011). Components of interference control predict depressive symptoms and rumination cross-sectionally and at six months follow-up. *J. Behav. Ther. Exp. Psychiatry* 42 65–73. 10.1016/j.jbtep.2010.06.001 20650447

[B54] Zhang J. X. Leung H. C. Johnson M. K. (2003). Frontal activations associated with accessing and evaluating information in working memory: an fMRI study. *Neuroimage* 20 1531–1539. 10.1016/j.neuroimage.2003.07.016 14642465

